# Long-term treatment with chloroquine increases lifespan in middle-aged male mice possibly via autophagy modulation, proteasome inhibition and glycogen metabolism

**DOI:** 10.18632/aging.204069

**Published:** 2022-05-23

**Authors:** Thorsten R. Doeppner, Cristin Coman, Daiana Burdusel, Diana-Larisa Ancuta, Ulf Brockmeier, Daniel Nicolae Pirici, Kuang Yaoyun, Dirk M. Hermann, Aurel Popa-Wagner

**Affiliations:** 1 Department of Neurology, University Medical Center Göttingen, Göttingen 37075, Germany; 2 Research Institute for Health Sciences and Technologies (SABITA), Medipol University, Istanbul, Turkey; 3 Department of Anatomy and Cell Biology, Medical University of Varna, Varna, Bulgaria; 4 Cantacuzino National Medico-Military Institute for Research and Development, Bucharest 050096, Romania; 5 Department of Biochemistry, University of Medicine and Pharmacy Craiova, Craiova 200349, Romania; 6 Faculty of Veterinary Medicine, University of Agronomic Sciences and Veterinary Medicine of Bucharest, Bucharest, Romania; 7 Vascular Neurology and Dementia, Department of Neurology, University of Medicine Essen, Essen 45147, Germany; 8 Experimental Research Center for Normal and Pathological Aging, ARES, University of Medicine and Pharmacy Craiova, Craiova 200349, Romania

**Keywords:** chloroquine, longevity, middle-aged mice, toxicity, autophagy, proteasome

## Abstract

Previous studies have shown that the polyamine spermidine increased the maximum life span in *C. elegans* and the median life span in mice. Since spermidine increases autophagy, we asked if treatment with chloroquine, an inhibitor of autophagy, would shorten the lifespan of mice. Recently, chloroquine has intensively been discussed as a treatment option for COVID-19 patients. To rule out unfavorable long-term effects on longevity, we examined the effect of chronic treatment with chloroquine given in the drinking water on the lifespan and organ pathology of male middle-aged NMRI mice. We report that, surprisingly, daily treatment with chloroquine extended the median life span by 11.4% and the maximum life span of the middle-aged male NMRI mice by 11.8%. Subsequent experiments show that the chloroquine-induced lifespan elevation is associated with dose-dependent increase in LC3B-II, a marker of autophagosomes, in the liver and heart that was confirmed by transmission electron microscopy. Quite intriguingly, chloroquine treatment was also associated with a decrease in glycogenolysis in the liver suggesting a compensatory mechanism to provide energy to the cell. Accumulation of autophagosomes was paralleled by an inhibition of proteasome-dependent proteolysis in the liver and the heart as well as with decreased serum levels of insulin growth factor binding protein-3 (IGFBP3), a protein associated with longevity. We propose that inhibition of proteasome activity in conjunction with an increased number of autophagosomes and decreased levels of IGFBP3 might play a central role in lifespan extension by chloroquine in male NMRI mice.

## INTRODUCTION

By the end of 2030 the number of people older than 60 will increase by 56% to reach 1.4 billion (WHO). However, the quality of life in the elderly depends heavily on their health conditions. Therefore, there is a need in finding treatments to enable aged people to live in good health to avoid the negative impact of poor health for society.

A popular method to increase lifespan and health conditions is calorie restriction (CR) both in lower organisms [1] and rodents [2, 3]. Thus, intermittent fasting at the beginning of adulthood induced lifespan expansion by 25% in *D. melanogaster* most likely by a mTOR-independent mechanism [1].

The effect of CR has been also studied in non-human primates whose physiology and life style are very similar to that of humans. One study conducted on Rhesus monkey (*Macaca mulatta*) at the University of Wisconsin reported a significant positive impact of CR on health, age-related survival [4], and all-cause survival [5], but the study conducted at the National Institute on Aging study detected no significant effect on longevity [6]. However, a direct comparison of longitudinal data from both studies has revealed differences in the source of the monkeys, feeding practices, diet composition, macronutrient composition and finally suggested that the age of onset of CR may be a critical factor that determines the extent of the beneficial effects of CR on health and longevity [7].

Recent studies involving energy metabolism and adipose tissue have provided novel insights into the possible mechanisms underlying longevity and health span. Indeed, nonshivering thermogenesis via enhanced mitochondrial uncoupling in the brown adipose tissue has been associated with increased longevity in CR animals and the dwarf mice [8, 9].

Many studies investigated the effects of CR-mimetics like resveratrol, a plant derivative with anti-oxidants and anti-inflammatory properties, on health- and lifespan in *S. cerevisiae*, *C. elegans* and *D. melanogaster.* In *C. elegans*, resveratrol did extend lifespan only under conditions of enhanced oxidative stress caused by high glucose concentrations in the culture medium [10]. In another study, treatment with resveratrol increased mean and maximum lifespan by delaying the onset of the “dying phase” [11].

In higher organisms, like mice, resveratrol treatment shifts the signaling pathways of middle-aged mice kept on a high-calorie diet towards that of mice on a standard diet and significantly increases their survival [12]. However, in other studies resveratrol given intraperitoneally to mice did not act as CR mimetic [13] suggesting that the effect of resveratrol on longevity in flies, worms or mice may vary from species to species. Further, resveratrol given as a food supplement to humans did not show positive results on the health status [14].

Inhibition of mTOR (target of rapamycin) signaling using rapamycin led to an extension of lifespan in *Caenorhabditis elegans* by 250% [15] and by 20% in *Drosophila melanogaster* [16]. In mice, treatment with rapamycin increased life expectancy by 10% in males and 18% in females [17]. Other beneficial effects of rapamycin include a dose-dependent decline in the liver degeneration in male mice, reduced number of atypical nuclei in the heart and tumors of the adrenal gland and improved tendon elasticity. Behaviourally, rapamycin delayed the age-related decline in spontaneous activity [17]. However, rapamycin treatment also led to a pronounced testicular tubular degeneration and cataracts in mice suggesting that time to start the treatment and the dosage should be carefully investigated [18].

In the clinic, rapamycin has been used as an immunosuppressant and through its anti-proliferative action, as a potential anticancer agent. However, long-term use as immunosuppressor may have side effects including gonadal dysfunction and infertility [19, 20], an effect that has been reported in aged male mice, too [18].

Another approach to extend life span is to enhance autophagy. Autophagy is a major pathway for the turnover of cell organelles and may play a pivotal role in health- and lifespan extension [21–24]. For example, long-term treatment of young and middle-aged mice with the autophagy enhancer spermidine led to an increased mean lifespan by 11% and extended longevity by 8% most likely by enhancing cardiac autophagy [25]. However, the effect of spermidine seems to be species-specific. For example, work done in our group has shown that in rats, autophagy enhancement by spermidine treatment increased the healthspan by attenuating neuroinflammation, anxiety and the exploratory behavior, but not the maximum life span [26]. On this occasion, we asked if the autophagy inhibitor chloroquine (CQ), would shorten the lifespan of rodents. Contrary to our hypothesis, we observed an extension, not reduction of lifespan by 11.7% by CQ when given in drinking water to middle-aged male NMRI mice. We therefore examined possible mechanisms underlying lifespan augmentation by chloroquine.

## RESULTS

### Treatment with chloroquine expanded the median and maximum lifespan in middle-aged male NMRI mice

**Figure 1:**
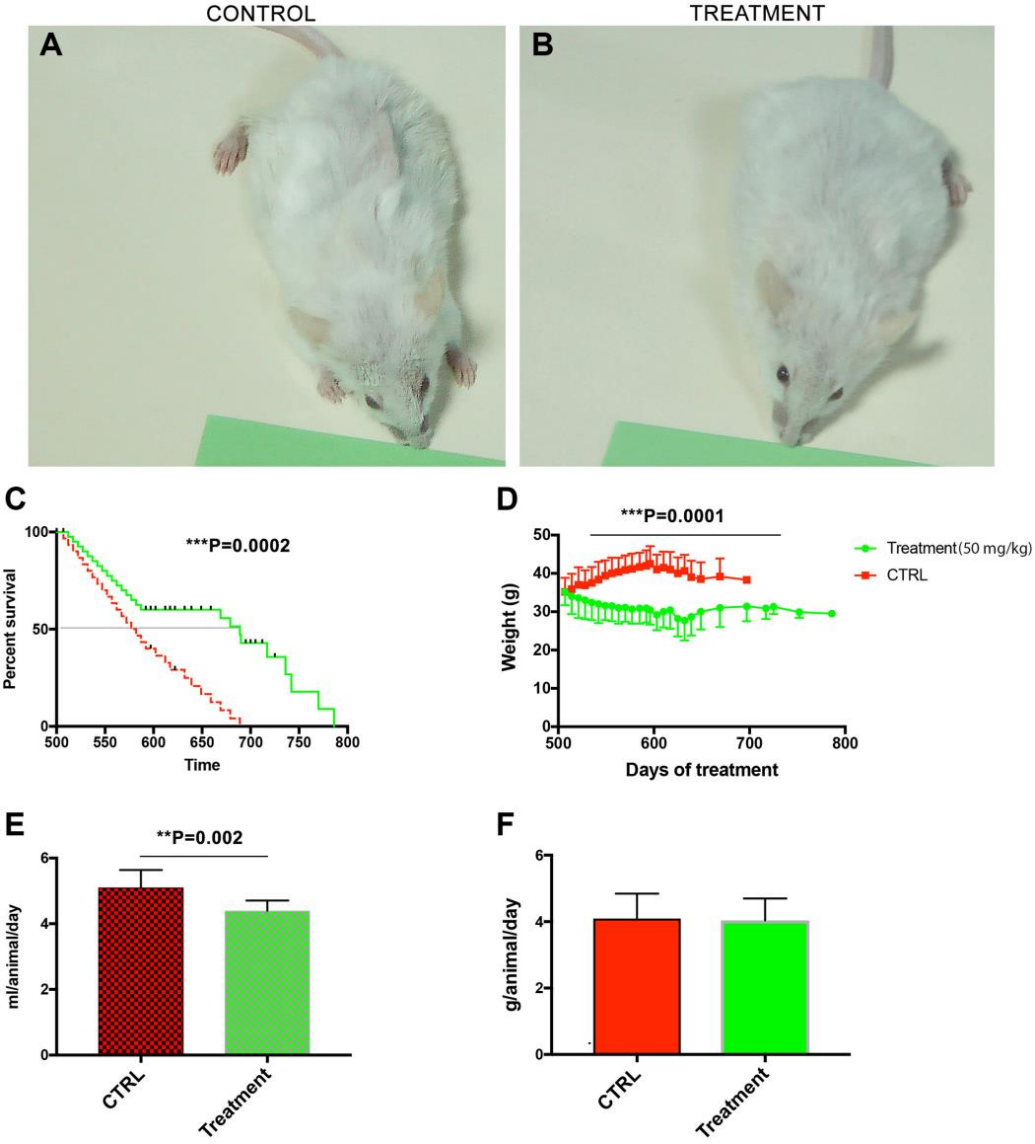
**Treatment with chloroquine expanded the median and maximum lifespan in middle-aged male NMRI mice.** Macroscopically, the fur of the treated animals looked less ruffled as compared to controls (**A** vs. **B**). The male mice fed with CQ significantly lived longer (786 days) than controls (689 days) and the median lifespan was also significantly different between the two groups (**C**). Long-term treatment with CQ (50 mg/kg) led to a gradual increase in the body weight of control animals that peaked at day 600 (*t*-test, two-tailed) (**D**). The volume of liquid intake was, on the average, significantly lower in the CQ-treated group (*t*-test, two-tailed) (**E**). However, the average amount of consumed food was not significantly different (**F**). Data are mean ± SD values. *N* = 28 for each group.

Treatment was initiated at the age of 500 days and continued for 286 days. Macroscopically, the fur of the treated animals looked less ruffled as compared to controls (Figure 1A vs. 1B). The male mice fed with CQ significantly lived longer (786 days) than controls (689 days) (*P* = 0.0002), and the median lifespan was also significantly different between the two groups (Figure 1C). When the treatment was initiated, the body weights were similar. However, in the control mice gradually there was an age-related increase in the body weight that was prevented by the CQ treatment (*P* = 0.0001; *t*-test, two-tailed) between the weight of the control mice and the weight of CQ-treated animals between days 600–700 (Figure 1D). The volume of liquid intake was, on the average, significantly lower (*P* = 0.002; *t*-test, two-tailed) in the CQ-treated group (Figure 1E). However, the average amount of consumed food was not significantly different between the two groups (Figure 1F).

### The lower dose of CQ did not cause significant pathological changes in the liver and the heart

**Figure 2:**
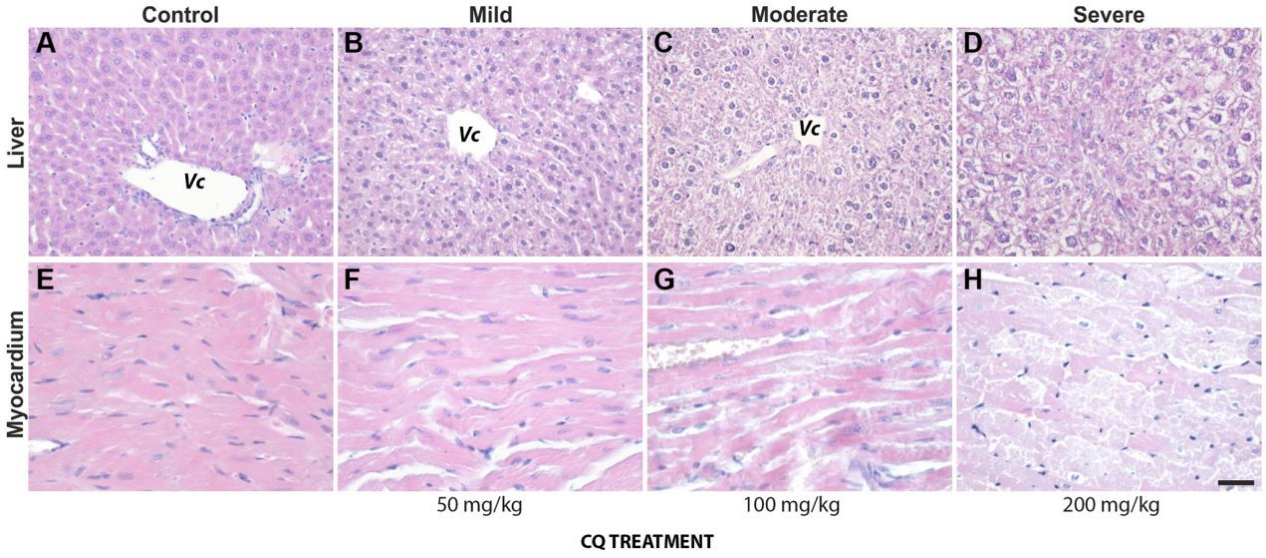
**The lower dose of 50 mg/kg CQ did not cause significant pathological changes in the liver and the heart.** (*upper panel*): Pathological changes in the liver of controls (**A**) and treatment (50 mg/kg) (**B**–**D**) consisted of hydropic degeneration and hepatocyte necrosis. The changes were visible mostly in portal areas and as they became more advanced, they spread through the lobule. Fibrosis and mononuclear inflammatory infiltrates were also present on occasion in portal spaces. (*lower panel*): In the heart of controls (**E**) and treatment (50 mg/kg) (**F**–**H**), interstitial oedema, intercalate disks fragmentation, loss of myocardiocyte striations and even areal necrosis could be seen, more frequent and more intense with increasing treatment dosage. Abbreviation: *Vc*, central vein. *N* = 10 for each group.

On autopsy, one control and two of the treated mice presented with retroperitoneal cysts. One control and one of the treated animals had a retroperitoneal tumor masses. In treated animals, at higher doses (100- and 200 mg/kg), however, the most frequent and obvious histological changes occurred in the liver, and consisted in hydropic degeneration and hepatocyte necrosis, both indicators of hepatocyte toxicity and intense metabolic distress. The changes were visible mostly in portal areas and as they became more advanced, they spread through the lobule. Fibrosis and mononuclear inflammatory infiltrates were also present on occasion in portal spaces rather than with a central or intralobular distribution, and thus were not included in the stratification of liver pathology (Figure 2, upper panel).

In the heart, interstitial oedema, intercalate disks fragmentation, loss of myocardiocyte striations and even areal necrosis could be seen, more frequent and more intense with increasing treatment dosage (Figure 2, lower panel). However, overall, we noted that the dose of 50 mg/kg of CQ did not cause significant pathological changes in the liver and heart (Figure 2; Table 1).

**Table 1:** Overall, we noted that the dose of 50 mg/kg of CQ did not cause significant pathological changes in the liver and heart.

**Liver**	**50 mg/kg**				**100 mg/kg**				**200 mg/kg**			
Pathological grading	GVD	PII	PF	HN	GVD	PII	PF	HN	GVD	PII	PF	HN
Mild (+)	1/10	0/10	1/10	2/10	3/10	2/10	0/10	2/10	3/10	3/10	2/7	5/10
Moderate (++)	3/10	1/10	0/10	1/10	4/10	0/10	0/10	1/10	2/10	1/10	0/7	5/10
Severe (+++)	1/10	0/10	0/10	1/10	3/10	0/10	0/10	0/10	2/10	0/10	0/7	0/10

Abbreviations: GVD: Granulovacuolar degeneration; PII: Portal inflammatory infiltrate; PF: Portal fibrosis HN: hepatocyte necrosis.

**Table:** 

**Heart**	**50 mg/kg**		**100 mg/kg**		**200 mg/kg**	
Pathological grading	IO	LS	IO	LS	IO	LS
Mild (+)	6/10	4/10	4/10	1/10	6/10	4/10
Moderate (++)	1/10	1/10	3/10	3/10	5/10	2/10
Severe (+++)	0/10	0/10	1/10	1/10	2/10	1/10

Abbreviations: IO: interstitial oedema; LS: loss of striations. *N* = 10 for each group.

### CQ treatment caused an increase in the LC3-II/LC3-I ratio and p62 levels in the liver and heart

Chloroquine at 60 mg/kg has been shown to disturb autophagy in mice by inducing an autophagy-independent severe disorganization of the Golgi and endolysosomal systems, which might contribute to autophagosome-lysosome fusion impairment and degradation of intra-autophagosomal components by lysosomal hydrolases [27].

Physiologically, lysosomal turnover of autophagosome reflects starvation-induced autophagic activity and the structural proteins of autophagosomes are routinely used to assess the effect of drugs on autophagy in animal models. The microtubule-Associated Protein 1 Light Chain 3 (LC3) family of proteins required for phagocytic clearance are the major structural proteins of autophagosomal membranes and consists of three highly homologous members, MAP1LC3A (LC3A), MAP1LC3B(LC3B), and MAP1LC3C (LC3C) [28].

**Figure 3:**
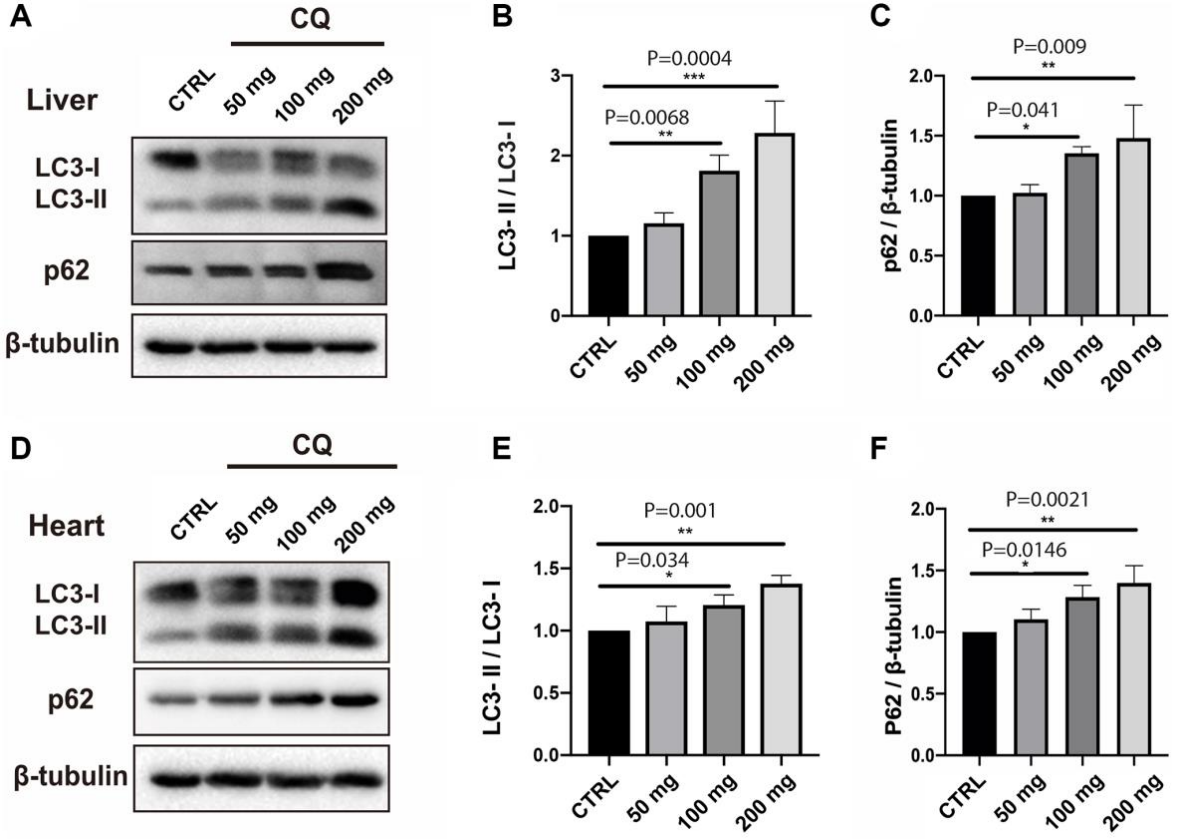
**CQ treatment caused an increase in the LC3-II/LC3-I ratio and p62 levels in the liver and heart homogenates.** In our toxicity study, there was a progressive, significant increase in the LC3-II/LC3-I ratio in the liver (**A**, **B**). The increase in the LC3-II/LC3-I ratio was paralleled by an increase in the expression level of p62 in the liver (**C**). The increase in the LC3-II/LC3-I ratio with increasing chloroquine concentration in drinking water was also significant, albeit less obvious in the heart homogenates (**D**, **E**). The increase in the LC3-II/LC3-I ratio was paralleled by an increase in the expression level of p62 in the heart (**F**) homogenates. Data are mean ± SD values. *N* = 5 for each group.

In our toxicity study, there was a progressive, significant increase in the LC3-II/LC3-I ratio in the liver (Figure 3A, 3B) [F(3,8) = 19.84; *P* = 0.0005] and to a lesser extent in the heart (Figure 3D, 3E) [F(3,8) = 12.69; *P* = 0.0021] homogenates with increasing chloroquine concentration in drinking water. Interestingly, CQ treatment significantly increased the expression of both LC3-I and LC3-II in the heart. However, there was a clear conversion of LC3-I to LC3-II in the liver of CQ-treated animals as compared to control animals.

p62 is a protein that interacts with autophagic substrates and delivers them to autophagosomes for degradation. The increases in LC3-II were paralleled by an increase in the expression level of p62 in the liver (Figure 3C) [F(3,8) = 8.32; *P* = 0.0077] and heart (Figure 3F) [F(3,8) = 10.97; *P* = 0.0033] homogenates with increasing chloroquine concentration in drinking water.

### LC3B immunohistochemistry

**Figure 4:**
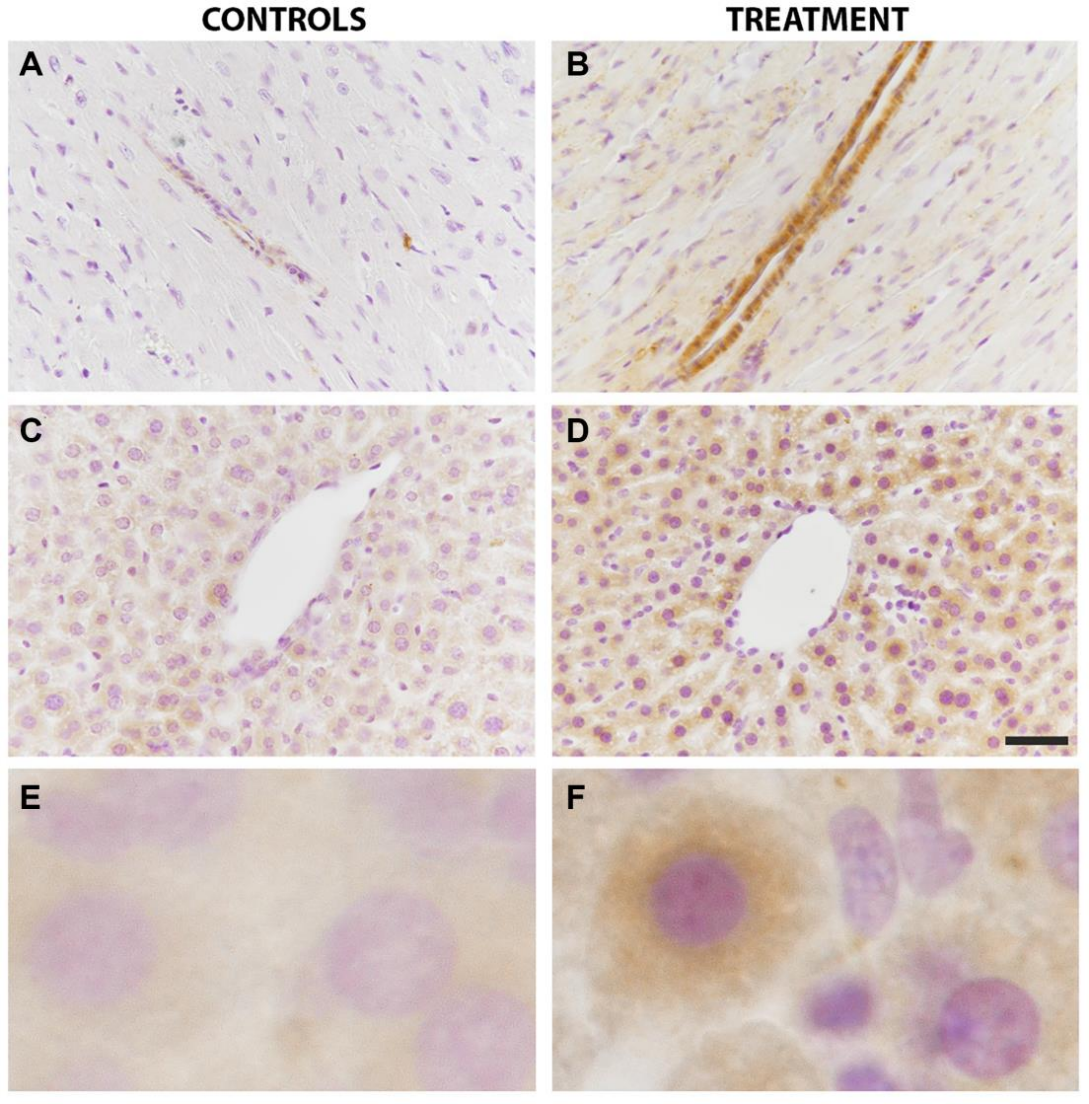
**LC3B immunoreactivity in the liver and heart was increased by chloroquine treatment.** In the heart tissue, a strong immunostaining was visible in the smooth muscle layer of both arterioles and venules from the parenchyma of treated animals as compared to controls (**A** vs **B**). In the liver, LC3B immunostaining showed a diffuse granular pattern in the cytoplasm of hepatocytes that was more intense in treated animals as compared to controls (**C** vs **D**). Enlarged images are shown for controls (**E**) and treatment (**F**). *N* = 10 for each group.

In the myocardium, the anti-LC3B antibody exhibited a very fine granular pattern in the cytoplasm of myocardiocytes. A strong immunostaining was visible in the smooth muscle layer of both arterioles and venules from the parenchyma of treated animals (Figure 4B) as compared to controls (Figure 4A).

In the liver, LC3B immunostaining showed a diffuse granular pattern in the cytoplasm of hepatocytes, without a preferred disposition in the lobules. LC3B immunostaining was more intense in treated animals as compared to controls (Figure 4C vs. 4D). Enlarged images of LC3B immunostainings in hepatocytes of controls and treated animals are also shown (Figure 4E vs. 4F).

### Electron microscopy

**Figure 5:**
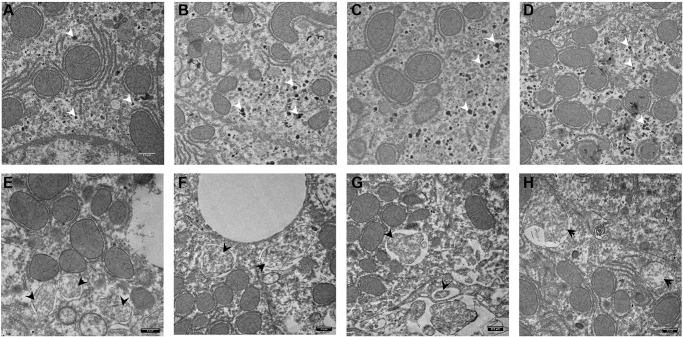
**Electron microscopy images of autophagosomes in mouse liver.** Control animal (**A**–**D**) vs. Chloroquine-treated animal (**E**–**H**). Highlighted are glycogen granules (white arrow) in controls and autophagosomes (black arrow) in the liver of treated animals. Scale bar, 0,5 μm. *N* = 3 for each group.

Four animals in each group were analyzed by EM. In the control group we could not see autophagosomes (Figure 5A–5D). Instead, there was a massive accumulation of glycogen granules in the liver cells (white arrowheads). Following CQ treatment, autophagosome-like structures that were encircled by double membranes containing portions of cytoplasmic organelles were detected (Figure 5E–5H; black arrowheads).

### Higher doses of CQ attenuated proteasome activity in the liver and heart

**Figure 6:**
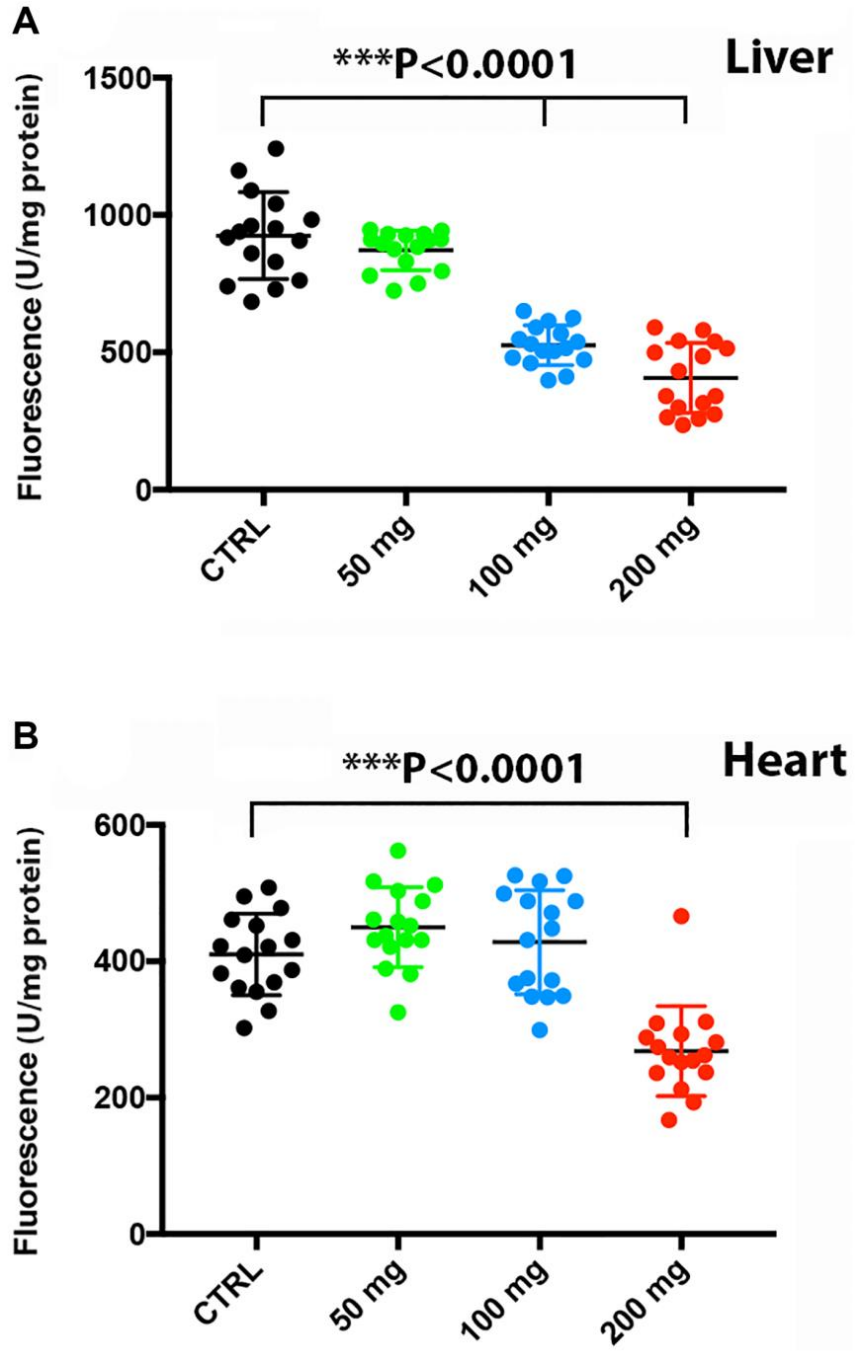
**Higher doses of CQ attenuated proteasome activity in the liver and heart.** Treatment with increasing doses of chloroquine significantly reduced proteasome activity in the liver at a dose of 100 mg/kg (**A**), whereas proteasomal activity in the heart was reduced only at 200 mg/kg (**B**). Data are given as arbitrary fluorescence units per mg protein as indicated in the materials and method section of the manuscript. *N* = 5 for each group.

There is an intimate linkage of proteostasis with the aging process and associated pathologies [29]. In addition, autophagy and proteostasis are interconnected [30]. We found that at a dose of 100 mg/kg and higher, proteasome activity was significantly reduced in the liver [F(3,45) = 63.2, *P* < 0.0001] (Figure 6A), whereas proteasomal activity in the heart was reduced only at the highest CQ dose of 200 mg/kg [F(3,45) = 24.24, *P* < 0.0001] (Figure 6B). Of note, treatment with 50 mg/kg did not reduce significantly the proteasomal activity in the liver and heart in the toxicity study.

### Serum insulin growth factor binding protein 3 was decreased by chloroquine treatment

**Figure 7:**
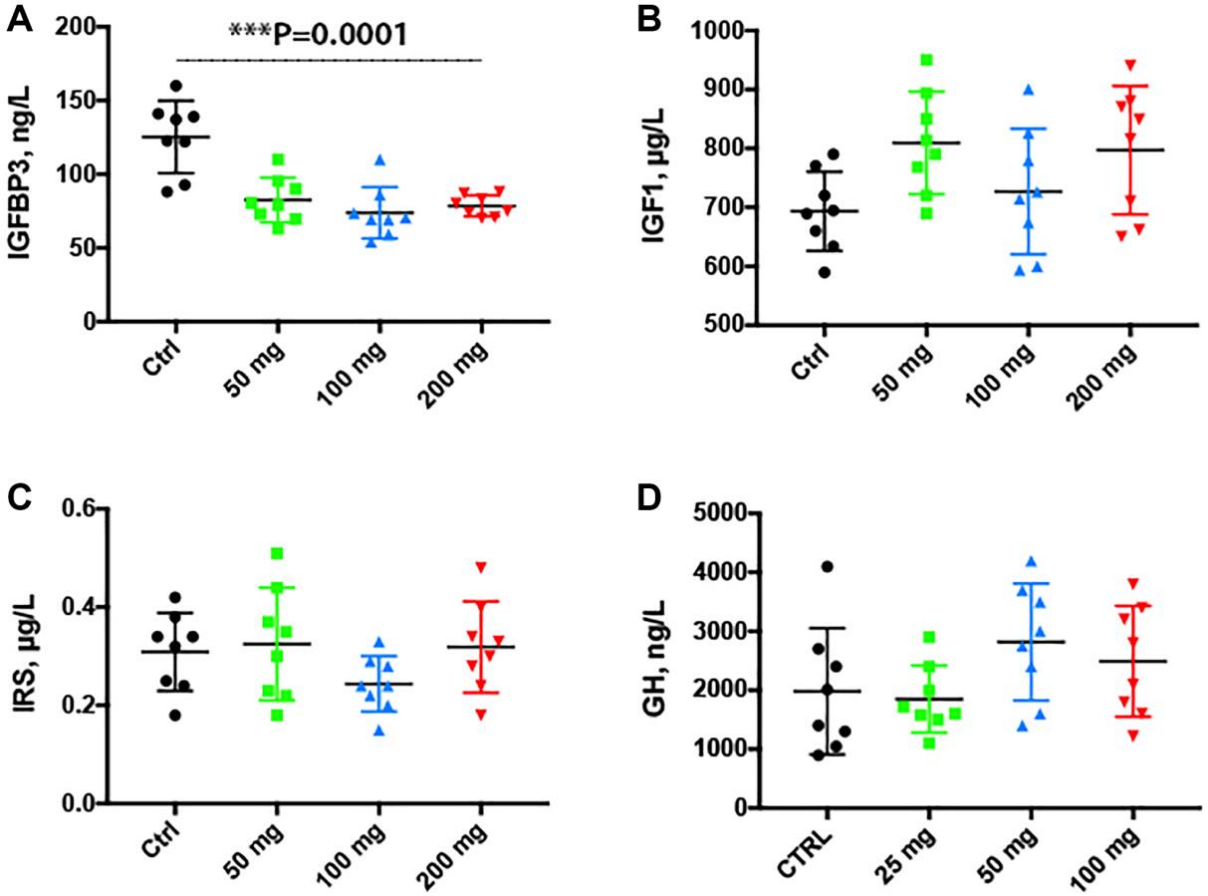
**Serum insulin growth factor binding protein 3 was decreased by the chloroquine treatment.** Treatment for 2 months with increasing doses of CQ did not cause significant changes in the body weight (**A**). Note that serum IGFBP3 was significantly decreased in serum collected from animals treated with CQ for 3 months (**B**). There was no statistically significant difference in the levels of serum IGF-1 and IRS of treated animals (**C**, **D**). Data are mean ± SD values. *N* = 15 for each group.

The IGF system plays an important role in regulating signal pathways involved in aging. By ELISA, we found that serum IGFBP3 was significantly decreased (*P* = 0.0001) in serum collected from animals treated with CQ for 3 months as compared to controls (Figure 7A). There was no statistically significant difference in serum IGF-1, IRS or GH levels of treated animals as compared to controls (Figure 7B–7D).

## DISCUSSION

Living a longer, healthier life is at the focus of aging research. Recently, administration of an autophagy enhancer, spermidine, in drinking water to pre-aged male and female mice significantly prolonged median lifespan by ~10% [25]. However, as reported by us, the same drug given to middle-aged male rats may not extend lifespan but healthspan by attenuating neuroinflammation and improving anxiety and exploratory behavior [26]. In this context, we asked if chloroquine, a drug which inhibits autophagy by disorganizing the Golgi complex and the endolysosomal system *in vitro* and *in vivo* [27], would shorten the lifespan in middle-aged male mice. We report that, surprisingly, chloroquine administered in drinking water at a dose of 50 mg/kg extended the median lifespan of middle-aged NMRI male mice by 11.8% and the maximum life span by 11.4%.

Aging mice normally develop a ruffled fur [31]. Macroscopically, the fur of the treated mice looked smoother than the fur of controls. Further, the mice in the treatment group did lose, on the average, 27% of their weight at 90 days of treatment. However, with increasing time, the difference in weight became less noticeable possibly due to decreasing numbers of survivors.

Except for a report on retinopathy after long-term use (250 mg CQ daily, for seven years) in humans [32], studies on CQ organ toxicity in young rats given at a dose of 124 mg/kg over a period of 6 weeks, showed marked hydropic degeneration and necrosis in the liver and heart [33]. In our experiments, 50 mg/kg given in drinking water over 12 weeks did not cause significant pathological changes in the heart. In the liver, mononuclear infiltrates were present occasionally in portal spaces, especially at higher CQ concentrations of 100 and 200 mg/kg.

Microtubule-associated protein light chain 3 (LC3-II) is generated by the conjugation of cytosolic LC3-I to phosphatidylethanolamine (PE) on the surface of nascent autophagosomes and is widely used to monitor autophagy [34, 35]. When CQ is given intraperitoneally at a dose of 60 mg/kg, it mainly inhibits autophagy by impairing autophagosome fusion with lysosomes [27].

In our toxicity study, we found, by Western blotting, a dose-dependent increase in the levels of the autophagy marker LC3-I/II in the liver and to a lesser extent in the heart, suggesting that indeed, CQ disrupted autophagy by impairing autophagosome fusion with lysosomes which led to the accumulation of LC3-II on autophagosomes which is, at least in part, attributable to an impaired autophagic flux [36]. The accumulation of LC3B-I/II was also confirmed on liver and heart tissue sections.

Studies from other groups question the simple relationship between LC3-I and LC3-II. Thus, it was found that autophagy inducing signals can stabilize LC3-I levels and subsequently increase LC3-II expression and increase autophagic flux, which means consistently induced autophagy may continue replenishment of LC3-II from the larger pool of LC3-I [37].

However, the increased LC3B immunoreactivity on liver and heart tissue could not be specifically attributed either to LC3B-I or LC3B-II because the antibody we have used did not distinguish between LC3B-I and LC3B-II. It should be noted that LC3B-positive puncta become larger and are prominent even with transient and incomplete inhibition of autophagosome biogenesis [35]. Moreover, CQ lipidation of LC3-I might not be related to autophagosome accumulation and highlights the need for greater understanding of the functional consequences of noncanonical autophagy [38].

TEM images reported by us also support the hypothesis that long-term treatment led to an accumulation of autophagosomes due to impaired autophagosome fusion with lysosomes.

Chloroquine has been shown to decrease proteolysis in human neuroblastoma SK-N-SH cells and in WT or Atg5−/− mouse embryonic fibroblasts [39]. Indeed, in our toxicity experiment, at higher doses of CQ, there was a decrease in the proteasome activity in the liver and heart tissue. Of note, the dose we have used for longevity studies did not change, after 60 days of treatment, the proteasome activity in the liver and heart. However, we cannot exclude that a longer treatment (9 months) might have reduced the proteasome activity in the liver and heart.

Regulation of proteasomal activity is a well characterized signaling pathway under both physiological and pathophysiological conditions [40]. A progressive decline in proteasome activity has been reported for the aging rat liver and heart suggesting either age-related changes in proteasome structure or increases in the level of oxidized and ubiquitinated proteins [41–43]. Although numerous reports indicate a decrease in proteasome activity during aging in animal models [44], new developments in the measurement of protein turnover indicate, except for the fat tissue, minimal changes in protein abundance in mouse tissues up to 26 months [45, 46].

The biological role of proteasomal activity, however, depends on cell and tissue conditions. As such, regulation of proteasome activity is a double-edged sword. Several studies have shown that proper proteasomal activity is essential for both cell cycle completion and cell survival [47]. In previous work, we have shown that proteasome inhibition was neuroprotective and was associated with increased post-stroke neurogenesis and angiogenesis that persisted at least for three months [48]. Herein, it is fair enough to assume that proteasomal inhibition may be in part responsible for the observed effects on longevity.

Growth hormone, insulin and the IGF (insulin-like growth factor) signaling pathway, all involved in the regulation of carbohydrate metabolism, play an important role in aging in animal models by activating multiple intracellular signaling cascades [49, 50]. Thus, inhibition of IGF signaling is thought to delay aging. However, the molecular mechanism underlying the modulation of lifespan by IGF signaling are still incompletely understood [51]. In our experiments, 2-months treatment with CQ lowered the levels of serum IGFBP3, a metabolic regulator that inhibits insulin-stimulated glucose uptake in murine models [52].

Autophagy mediates the degradation of cellular components in lysosomes and the resulting products are used for synthetic processes. The remaining, mostly fatty acids and to a lesser extent amino acids, are used to generate energy [53]. Accumulation of glycogen granules in controls suggests that CQ interferes with glycolysis. A similar observation has been reported in Zmpste24−/− mice, progeria mouse model [54]. A reduction in substrate availability of the components of the Krebs cycle following CQ treatment has been previously reported [55]. We hypothesize that the reduction in energy availability caused by the blockade of fusion of autophagosomes to the lysosomes, is compensated by an increased degradation of glycogen that could not be seen in the treated animals.

Previous studies have shown that *in vitro*, IGFBP3 is increased in aging human fibroblasts [56] and human umbilical endothelial cells from aged humans [57]. In animal studies, aging in rats has been associated with decreased levels of GH and IGFBP3 in liver and serum [58]. Therefore, one of the beneficial effects of the CQ treatment was indeed, to lower the levels of serum IGFBP3. However, the treatment did not significantly change the levels of serum GH, IGF1 and IRS.

CQ was originally discovered and used to prevent or treat malaria and amebiasis [59], and subsequently inflammatory diseases [60, 61]. CQ and its derivate hydroxychloroquine (HCQ) are FDA-approved drugs and are currently the principal compounds used in clinical trials aimed to treat tumors through autophagy inhibition [62].

Autophagy eliminates long-lived proteins, insoluble protein aggregates and even mitochondria, peroxisomes and bacteria whereas the ubiquitin–proteasome system (UPS) is responsible for the degradation of short-lived proteins and soluble misfolded proteins [63]. The UPS and autophagy are interconnected. Thus, inhibition of one system led to a compensatory upregulation of the other system in order to maintain cellular homeostasis [30, 64].

Furthermore, HCQ given *ip* to mice at a dose of 60 mg/kg induced an autophagy-independent severe disorganization of the Golgi and endo-lysosomal systems in kidney and intestinal cells calling for caution when interpreting results obtained by blocking autophagy with this drug [27]. Indeed, the action of CQ on the cell seems to be dose-dependent. Thus, treatment of Zmpste24−/− mice (a progeria mouse model with low DNA repair capacity) with CQ given in 0.9% saline twice per week at 3.5 mg/kg body weight for about 3 months, activates Ataxia telangiectasia mutated (ATM), a serine/threonine protein kinase, a key regulator of DNA damage response, promotes DNA damage clearance, ameliorates premature aging and extends lifespan by 19% [54]. However, the effect of CQ in the wild type mice has not been shown. Moreover, CQ treatment did not extend lifespan in Atm−/− mice. Therefore, we hypothesize that the initial target of CQ could be the proteasome system those inhibition could lead to the upregulation of LC3B-II on the autophagosomes.

## CONCLUSION

Our results suggest that chloroquine extends the maximal lifespan of middle-aged mice possibly by disrupting autophagocytosis, decreasing proteolysis and increasing glycogen metabolism in the liver and heart in male NMRI mice. Systemically, we measured decreased levels of serum IGFPB3. Given the constancy of the body weight in CQ-treated animals might also imply that the effects of CQ in treated mice could be similar to caloric restriction or possibly through an effect on amino acid homeostasis. Indeed, a wide-scale comparative analysis of longevity genes and interventions indicates a quite ambiguous role of autophagy in the control of longevity [65]. Quite intriguingly, chloroquine treatment was also associated with a decrease in glycogenolysis in the liver suggesting a compensatory mechanism to provide energy to the cell that supports the concept of “physiological hormesis” that might have been caused by CQ-induced mild stress in the liver [66]. Clearly, further studies are needed to explore the underlying mechanisms at several chloroquine doses and to extend the treatment to female mice and rats.

## MATERIALS AND METHODS

### Animals and treatment

Middle-aged (500 days old) NMRI (Naval Medical Research Institute) mice were kept on a 12-hour light/dark cycle at 23°C, having free access to standard food and water. The mice were randomly assigned to two groups: (1) control group (*N* = 28) and (2) treatment group (*N* = 28). Mice were treated with 50 mg/kg chloroquine (CQ; Sigma-Aldrich, Munich, Germany) dissolved in drinking water until death. The control group received water only. Survival was daily controlled. All studies on laboratory animals were performed in accordance with the Directive 2010/63/EU of the European Parliament and the Council of 22 September 2010 on the protection of animals used for scientific purposes with relevant acts and regulations. All protocols were approved by the local Animal Ethics Committee (#112-14112018), and all appropriate measures were taken to minimize pain and suffering.

### Chloroquine toxicity, histology, immunohistochemistry and electron microscopy

For toxicity studies CQ (3-months old mice; 50, 100, and 200 mg/kg body weight; *N* = 10) was administered in the drinking water. Control animals (*N* = 10) received water without CQ. After 2 months of treatment, animals were anesthetized with a mix of xylazine/ketamine and blood was collected by cardiac puncture. After intra-cardiac perfusion with 4% paraformaldehyde (PFA) and dissection, the organs were fixed for 48 h in 4% PFA and processed for paraffin embedding. Four micrometer-thick sections were cut and collected on poly-lysine coated slides. A first series of slides were routinely stained with hematoxylin and eosin-stained for diagnostic purposes.

For immunohistochemistry, slides were deparaffinated and re-hydrated, processed for antigen retrieval by boiling in citrate buffer pH6, endogenous peroxidase blocked in 0.1% water peroxide and unspecific binding sites blocked with skimmed milk. The primary antibody was added overnight (rabbit anti-LC3B, monoclonal, clone 12K5, 1:10; Sigma-Aldrich), and the next day the signal was detected with species-specific peroxidase-labelled polymer secondaries (Vector Laboratories) for 1 hour, then the sections were counterstained with hematoxylin and coverslipped. All sections have been imaged under a Nikon 90i microscope equipped with a plane-apochromat objectives and a Nikon DS-Ri2 16Mp CMOS camera.

We have assessed and graded histopathological changes in the liver and myocardium in all animal groups. For the liver, we have assessed the extent of hepatocyte hydropic degeneration and necrosis as absent (0), mild (+), moderate (++) or severe (+++). Also, using the same tier, we have evaluated interstitial myocardial oedema, loss of myocardiocyte striations and necrosis.

#### Electron microscopy

For transmission electron microscopy we treated 18-months old male mice with 50 mg/kg CQ for 2 months. Samples of liver from controls (*N* = 4) and treatment (*N* = 4) were fixed in 2.5% glutaraldehyde in 0.1 M PBS at 4°C overnight. The samples were subsequently post-fixed in 1% osmium tetroxide and further processed by standard procedures, including dehydration, infiltration and polymerization in araldite. Ultramicrotomy and transmission electron microscopy (TEM) was performed at the Electron Microscopy Unit (EMU) of the Imaging Center Essen (IMCES). Here, ultrathin sections with a diameter of 55 nm were generated using a Leica UC7 ultramicrotome and sections were collected on 200 mesh copper grids. After air drying, the samples were examined with a JEOL JEM-1400Plus, operating at 120 kV and equipped with a 4096 × 4096 pixels CMOS camera (TemCam-F416, TVIPS, Gauting, Germany).

### Western blot analysis of the autophagosomal membrane protein LC3B

Tissue lysates from heart and liver and lung (25 μg protein/well) were treated with sample buffer (dithiothreitol, 0.1% SDS, 0.1 M Tris HCl; pH 7.0) and boiled for 5 min at 95°C before separation on 12% SDS-polyacrylamide gel electrophoresis gels. The samples were then transferred to polyvinylidene fluoride membranes (Merck Group, Darmstadt, Germany). The membranes were blocked in 5% milk diluted with Tris-buffered saline solution with 1% Tween-20 for 1 h at room temperature and probed with LC3BI/II (1:1000, Abcam, Cambridge, UK), p62 and ß-tubulin (1:1000, Abcam, Cambridge, UK) antibodies overnight followed by 1 h of incubation with a matched horseradish peroxidase-labeled secondary antibody. Immunoreactivity was detected using chemiluminescence detection kit reagents and a Chemidoc Station (Bio-Rad, Hercules, CA, USA). Western blots from three independent experiments were quantified using ImageJ software (version 1.41, National Institutes of Health). To enhance the visibility of bands, images were equally adjusted for contrast in Adobe Photoshop.

### Analysis of proteasome activity

Liver and heart tissue were homogenized in a lysis buffer containing 100 mM Tris-HCl, 145 mM NaCl, 10 mM EDTA, and 0.5% Triton X-100 at pH 7.5 as previously described by us [27]. Chymotrypsin-like activity was evaluated by fluorescence in a reaction buffer consisting of 50 mM Tris, 20 mM KCl, 1 mM magnesium acetate, 2 mM dithiothreitol, 1 mM leupeptin (Sigma-Aldrich), 1 mM phenylmethylsulfonyl fluoride (PMSF; Merck) and Suc-Leu-Leu-Val-Tyr-AMC (Sigma-Aldrich; 50 μM) as a substrate. Proteasome activity was fluorimetrically measured at λ_exc._ 355 nm and at λ_em._ 460 nm and is given in arbitrary fluorescence units per mg protein. Protein concentrations were determined using the Bradford assay.

### Blood biochemistry

Growth hormone (GH; Cloud-Clone Corp. Wuhan, China), insulin-like growth factor 1 (IGF-1; RayBio, Norcross, GA, USA), insulin-like growth factor binding protein-3 (IGFBP-3; RayBio, Norcross, GA, USA) and insulin receptor substrate-1 (IRS-1; Cloud-Clone Corp. Wuhan, China) were determined in serum of controls and treated animals by ELISA following the manufacturer’s instructions.

### Statistical analysis

The effect of treatment on body weight, liquid intake was done using unpaired two-tailed *t* tests. The main effect of treatment on longevity was analyzed using Mantel-Cox test (GraphPad Software, San Diego, CA, USA). Statistical analysis regarding ELISA, Western blot and proteasome activity involved a one-way ANOVA followed by Tukey’s post-hoc test. Quantitative data were expressed as mean ± standard deviation (SD). *P* < 0.05 was regarded as statistically significant.

### Availability of data and material

Data will be made available to qualified researchers upon request.
